# Neonatal-lethal dilated cardiomyopathy due to a homozygous *LMOD2* donor splice-site variant

**DOI:** 10.1038/s41431-022-01043-8

**Published:** 2022-01-26

**Authors:** Michaela Yuen, Lisa Worgan, Jessika Iwanski, Christopher T. Pappas, Himanshu Joshi, Jared M. Churko, Susan Arbuckle, Edwin P. Kirk, Ying Zhu, Tony Roscioli, Carol C. Gregorio, Sandra T. Cooper

**Affiliations:** 1 Kids Neuroscience Centre, The Children’s Hospital at Westmead, Westmead, NSW Australia; 2 Discipline of Child and Adolescent Health, Sydney Medical School, University of Sydney, Camperdown, NSW Australia; 3 Department of Medical Genomics, Royal Prince Alfred Hospital, Camperdown, NSW Australia; 4 Department of Cellular and Molecular Medicine and Sarver Molecular Cardiovascular Research Program, The University of Arizona, Tucson, AZ USA; 5 Department of Histopathology, The Children’s Hospital at Westmead, Westmead, NSW Australia; 6 New South Wales Health Pathology, Randwick Genomics Laboratory, Randwick, NSW Australia; 7 School of Women’s and Children’s Health, University of New South Wales, Randwick, NSW Australia; 8 Centre for Clinical Genetics, Sydney Children’s Hospital, Randwick, NSW Australia; 9 Neuroscience Research Australia (NeuRA), University of New South Wales, Sydney, NSW Australia; 10 The Children’s Medical Research Institute, Westmead, NSW Australia

**Keywords:** Genetics research, Disease genetics, Functional genomics

## Abstract

Dilated cardiomyopathy (DCM) is characterized by cardiac enlargement and impaired ventricular contractility leading to heart failure. A single report identified variants in leiomodin-2 (*LMOD2*) as a cause of neonatally-lethal DCM. Here, we describe two siblings with DCM who died shortly after birth due to heart failure. Exome sequencing identified a homozygous *LMOD2* variant in both siblings, (GRCh38)chr7:g.123656237G > A; NM_207163.2:c.273 + 1G > A, ablating the donor 5′ splice-site of intron-1. Pre-mRNA splicing studies and western blot analysis on cDNA derived from proband cardiac tissue, MyoD-transduced proband skin fibroblasts and HEK293 cells transfected with *LMOD2* gene constructs established variant-associated absence of canonically spliced *LMOD2* mRNA and full-length LMOD2 protein. Immunostaining of proband heart tissue unveiled abnormally short actin-thin filaments. Our data are consistent with *LMOD2* c.273 + 1G > A abolishing/reducing *LMOD2* transcript expression by: (1) variant-associated perturbation in initiation of transcription due to ablation of the intron-1 donor; and/or (2) degradation of aberrant *LMOD2* transcripts (resulting from use of alternative transcription start-sites or cryptic splice-sites) by nonsense-mediated decay. *LMOD2* expression is critical for life and the absence of LMOD2 is associated with thin filament shortening and severe cardiac contractile dysfunction. This study describes the first splice-site variant in *LMOD2* and confirms the role of *LMOD2* variants in DCM.

## Introduction

Dilated cardiomyopathy (DCM) is characterized by enlargement of the heart, with left or biventricular dilation, and normal or reduced thickness of the left ventricular wall [1], leading to impaired ventricular contractility. The clinical course is commonly progressive and may result in heart failure if left untreated [1].

A monogenetic basis accounts for 25–50% of DCM cases [2, 3] and variants in more than 50 genes have been reported to be associated with DCM (reviewed in [4]). Genes associated with DCM as the predominant phenotype include *TTN* (Titin, 20–25% of cases), *LMNA* (Lamin A/C, ~5% of cases), *MYH7* (Myosin heavy chain 7, ~4% of cases), *TNNT2* (Troponin T, ~2% of cases), *MYBPC3* (Myosin-binding protein C, ~2% of cases), *MYPN* (Myopalladin, ~2% of cases), *SCN5A* (Sodium channel α unit, ~2% of cases), and *PLN* (Phospholamban, 1% of cases) [3]. Of these, multiple DCM-associated genes encode for structural proteins of the cardiac sarcomere (e.g., *TTN, MYH7, TNNT2, MYBPC3, MYPN*).

*LMOD2* encodes leiomodin-2 (LMOD2) which is expressed exclusively in striated muscle and is the predominant leiomodin in cardiac muscle [5]. Leiomodins (LMOD1, LMOD2, and LMOD3) are members of the tropomodulin superfamily and bind the pointed-end of actin-thin filaments. A homozygous nonsense variant in *LMOD2* (c.1193G > A, p.Trp398*) was previously identified in one individual affected with DCM, associated with absence of LMOD2 protein, severe shortening of thin filaments in the left ventricle, and contractile force deficiency in patient myocytes [6]. Likewise, *Lmod2* knock-out (KO) mice also present with early-onset, pre-weaning lethal DCM associated with cardiac muscle thin filament shortening [7, 8]. Homozygous or compound heterozygous variants in *LMOD3*, the predominant isoform expressed in skeletal muscle, are associated with severe-lethal nemaline myopathy associated with skeletal muscle thin filament shortening [9]. A homozygous nonsense variant in the smooth muscle specific isoform-*LMOD1* is linked to megacystis microcolon intestinal hypoperistalsis syndrome [10].

Herein we describe two infant siblings presenting with lethal, neonatal-onset DCM, associated with a homozygous variant ablating the 5’-splice-site of *LMOD2* intron-1 (GRCh38)chr7:g.123656237G > A; NM_207163.2:c.273 + 1G > A). This variant is the first pathogenic splice-site variant identified in *LMOD2*, providing important additional evidence supporting the association of *LMOD2* loss-of-function variants with severe DCM and cardiac thin filament shortening.

## Methods

### Massively parallel sequencing (MPS), Sanger sequencing

MPS of the exome of both parents and proband III:4 was undertaken at the NSW Health Pathology Randwick Genomics Laboratory, Sydney. Library preparation was performed using an Agilent SureSelect XT Low Input Clinical Research Exome x 2 (Agilent CRE v2) kit, with libraries analyzed on an Illumina NovaSeq 6000 instrument. A NATA-approved in-house pipeline (GAIA) was used to annotate, filter and prioritize high-quality variants that differed from the reference sequence [11]. The following parameters where then analyzed in identified candidate variant: mutational class, zygosity/inheritance patterns, population frequency, allele case frequency data (from disease-specific publications and ClinVar), conservation data, protein/domain structure databases, phenotype databases, pathogenicity predictions, functional/animal model studies, and mutation spectrum of the disease. Variants were de-prioritized if they had tolerant *in silico* scores, were predicted to have a low impact on protein structure or function, or if they occurred in the Genome Aggregation Database and internal laboratory databases for a fully penetrant Mendelian disorder. Variants were reviewed for their quality by inspecting them in Integrative Genomics Viewer [12]. Bi-directional Sanger sequencing was used to determine variant segregation in affected (III:3) and unaffected (III:1, III:2 and III:5) siblings.

### Cell culture, transfection, and transduction

Human skin fibroblasts from proband III:4 and an adult control were cultured in 1:1 Dulbecco’s modified Eagle medium/Nutrient Mixture F-12 (DMEM/F12, Gibco) containing 10% (vol/vol) fetal bovine serum (FBS; HyClone), 10% AmnioMax^TM^-II (Gibco) and 50 µg/ml gentamicin (Sigma). Muscle-specific genes expression was induced by transduction with a *MYOD1* adenovirus (Vector Biolabs, Adv-MyoD; see Supplementary Fig. I for details) [13].

Human embryonic kidney 293 (HEK293) cells were cultured in DMEM (11995-065) supplemented with MEM non-essential amino acids (11140-050), Pen-Strep (all Gibco) and 8% FBS (Omega Scientific, FB-02). Cells were transfected using Lipofectamine 3000 (Invitrogen), according to the manufacturer’s instructions, with pcDNA3.1(−) containing the *LMOD2* gene sequence (lacking 5′UTRs; corresponding to GRCh38.p13; chr7:g.123655964– g.123664441) cloned from human genomic DNA; or the *LMOD2* gene sequence containing the NM_207163.2:c.273 + 1G > A variant introduced via site-directed mutagenesis (two separate sequenced-verified mutant clones). Cells were harvested 48–72 h after transfection.

### RNA extraction, cDNA synthesis, and reverse transcription PCR (RT-PCR)

Cardiac tissue: RNA and protein were extracted from 30 × 8 μm thick muscle cryosections (~10 mm^2^ surface area). Tissue sections were collected in an eppendorf tube and 1 ml TRIzol™ (Invitrogen™) reagent and a 5 mm stainless steel bead were added followed by vigorous agitation in a QIAGEN TissueLyser (30 Hz, twice for 2 min). RNA and protein fractions were extracted according to the product guide. The RNA fraction was further purified using the RNeasy® Mini Kit (QIAGEN) and cDNA was synthesized from 385 ng RNA (Invitrogen SuperScript IV First-Strand Synthesis System) as described in the manufacturer’s protocol. PCR for <1 kb amplicons was performed using Buffer D (Astral Scientific), 1 Unit Taq DNA Polymerase (Life Technologies) and 0.3 mM each forward/reverse primer. PCR products of >1 kb were amplified using LongAmp® Taq DNA Polymerase (New England Biolabs) as per the manufacturer’s protocol.

HEK293 cells and MyoD-transduced skin fibroblasts (MyoD-fibroblasts): cells were washed twice with PBS and scraped off culture plates into RLT lysis buffer (QIAGEN) supplemented with 10 μl ß-mercaptoethanol. Cells were homogenized using a 20-gauge needle and processed using the RNeasy® Mini Kit (QIAGEN), including an on-column DNAse digestion for 15 min. cDNA was synthesized from 500 ng or 4000 ng total RNA using the Maxima First Strand cDNA Synthesis Kit for RT-qPCR (Thermo Scientific). All RT-PCR reactions were performed with cDNA corresponding to 10 ng of RNA, except for the *LMOD2* exon 1-2 reaction where 400 ng was used. GoTaq® DNA Polymerase (Thermo Fisher Scientific) was utilized for all RT-PCR reactions, except for reaction *LMOD2* Ex1-3 in which LongAmp® DNA Polymerase (New England Biolabs) was used.

PCR cycling conditions and primer details are described in Supplementary Methods and Supplementary Table I.

### Protein extraction and western blot analysis

Cardiac tissue: TRIzol™ protein fractions and frozen cardiac tissue sections from additional controls (ten sections of 8 μm thickness) were solubilised in Tris-SDS buffer (62.5 mM Tris-HCl pH 6.8, 4% sodium dodecyl sulfate (SDS), 1 × protease inhibitor cocktail (Sigma-Aldrich)), sonicated and heated for 4 min at 94 °C. Protein concentrations were then determined via a bicinchoninic acid assay as per manufacturer’s instructions (Pierce, Thermo Fisher Scientific). One part lysate was then mixed with three parts loading buffer (62.5 mM Tris-HCl pH 6.8, 4% SDS, 0.2 M DTT, 40% Glycerol, traces of bromophenol blue and 1 × protease inhibitor cocktail). Samples were separated using a 1-mm-thick, 4–12% SDS-polyacrylamide gel (NuPAGE™ Novex Bis-Tris precast gels, Life Technologies) followed by transfer onto a PVDF membrane (Merck Millipore Immobilon™-P, 0.45 µm) for 1 h in Tris-Glycine-buffer containing 0.075% SDS and 15% methanol. Membranes were probed with either anti-LMOD2 (1:500, Santa Cruz, sc-135493, S-12) or anti-leiomodin-3 (LMOD3) (1:10,000, Proteintech 14948-1-AP) as well as anti-cardiac actin antibodies (Ac1-20.4.2, PROGEN Biotechnik GmbH, Germany) as described previously [9] followed by total protein staining with Coomassie Blue Brilliant (Sigma-Aldrich).

HEK293 cells and MyoD-fibroblasts: cells were washed twice with PBS and scraped off culture plates into cold lysis buffer (25 mM HEPES pH 7.4, 150 mM NaCl, 1.5 mM MgCl_2_, 1 mM EGTA, 10 mM sodium pyrophosphate, 10 mM sodium fluoride, 0.1 mM sodium deoxycholate, 1% Triton X-100, 1% SDS, 10% (vol/vol) glycerol, 1x Halt protease inhibitor). Cell lysates were sonicated, and spun at 16,000 × *g* for 15 min at 4 °C. Protein concentrations were determined as described above and samples were boiled in 1x Laemmli sample buffer at 90 °C for 5 min. Lysate was resolved on a 10% or 15% SDS-polyacrylamide gel and transferred to an Immobilon-FL PVDF transfer membrane (0.45 μm, Thermo Fisher Scientific). Membranes were stained with Ponceau S followed by probing as described previously [14]. Primary antibodies included rabbit polyclonal anti-Lmod2 (1:1000, E13, Santa Cruz Biotechnology, Texas), mouse monoclonal anti-GAPDH (0.9 μg/ml, 6C5; Life Technologies), mouse monoclonal anti-MYH1E (1:50, MF201, Developmental Studies Hybridoma Bank), and rabbit polyclonal anti-LMOD3 (0.16 μg/ml, 14948-1-AP, Proteintech). Secondary antibodies included Alexa Fluor 680 or Alexa Fluor 790 AffiniPure donkey anti-rabbit or mouse immunoglobulin G (1:40,000; Jackson ImmunoResearch). Blots were imaged and analyzed using the Odyssey CLx imaging system (LI-COR Biosciences).

### Immunohistochemistry and thin filament measurements

Cardiac tissue was processed and sectioned as described in [14] followed by immunostaining as described in [6]. TMOD1 staining was used to measure thin filament lengths using ImageJ and was compared to a healthy control (female, 14 month).

## Results

### Clinical description

**Fig. 1:**
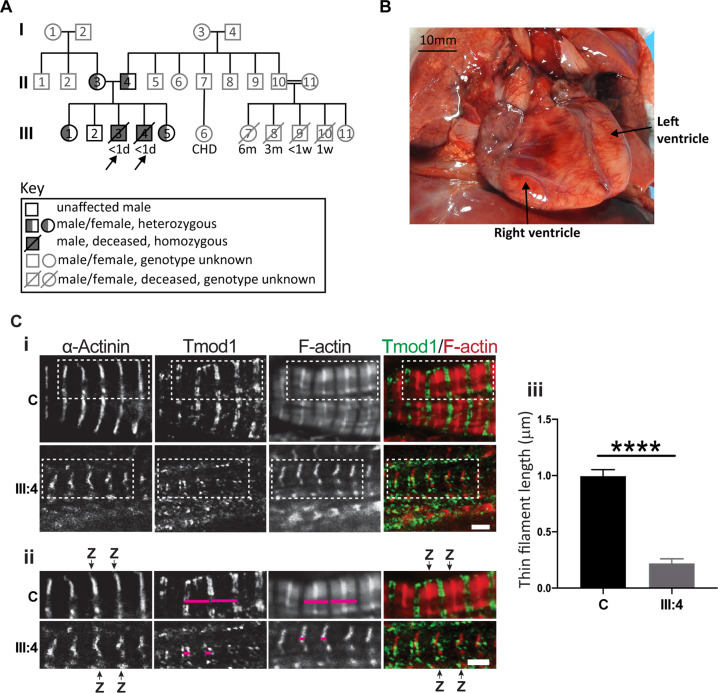
Family pedigree and heart pathology of III:4. **A** Pedigree of index family with proband III:3 and III:4 marked by arrows. Both probands are homozygous for the *LMOD2* c.273 + 1G > A variant (filled symbol) while unaffected immediate family members were either heterozygous (half-filled symbol) or did not carry the variant (unfilled symbol). A paternal uncle of the proband and his wife (II:10, II:11; cousins, consanguinity indicated by double line) had 5 offspring (III:7-11). Gray symbols: Genetic testing and medical history not available. Age at death is indicated underneath the symbol. **B** Anterior view of the heart of III:4 in situ demonstrating cardiac enlargement, in particular the left ventricle. Scale bar = 10 mm. **C** Immunostaining using anti-α-actinin antibodies (Z-disc), anti-Tmod1 antibodies (thin filament pointed-end; green) and fluorescent-conjugated phalloidin (F-actin, red) showed disorganized myofibrils and drastically shortened thin filaments in III:4 heart tissue compared to a 14-month-old non-failing control heart (C; NF14). Z-disc (Z) location is marked by black arrows and magenta lines designate thin filament arrays extending from the Z-disc. (ii) Enlargements of areas in white dash-line boxes of (i). Scale bar = 2 μm. (iii) Bar graphs showing shortened thin filament lengths in III:4 heart tissue versus the NF14 control. NF14 measures are consistent with previously published thin filament lengths [6] (Mean ± SD, *n* = 99–100 measurements, ****p < 0.0001, Student’s *t* test). d day, w week, m month, CHD congenital heart disease.

Family history: the index family includes three healthy children (III:1, III:2, III:5) and two affected male children (III:3 and III:4; third and fourth pregnancy) (see pedigree, Fig. 1A). The mother (II:3) and father (II:4), who originate from the same village in Egypt, are healthy and not known to be consanguineous. A paternal uncle (II:7) who lives in Egypt had a female child (III:6) with a congenital heart defect requiring surgery (III:6). A second uncle in Egypt (II:10) and his consanguineous wife (II:11, first cousins) had five children, with four infants dying in the first 6 months of life of unknown causes (III:7– III:10) and one healthy female child (III:11).

III:3: the pregnancy of III:3 was uneventful except for 2 weeks of reduced fetal movements after 36 weeks gestation. Cardiotocography and ultrasound investigations were normal, labor was induced, and a boy, III:3, was born by vaginal delivery at 38 weeks and 6 days gestation. III:3 appeared well at birth with Apgar scores of 9 at 1 and 5 min and a birth weight of 3780 g (86th percentile). He was noted to be grunting and cyanosed 1 h after birth and he was admitted to the neonatal intensive care where mechanical ventilation was initiated. Echocardiogram showed decreased ventricular function and chest X-ray detected cardiomegaly. On clinical examination, III:3 had hepatomegaly and globally weak pulses. Despite good ventilation, intravenous fluids and prostaglandin E1, his oxygen saturation did not improve above 50% during transport. Umbilical venous blood gas showed severe metabolic acidosis. On arrival at the tertiary intensive care unit, III:3 was flaccid with fixed pupils. Echocardiogram showed immobile bi-ventricular function with a large left-sided ventricular thrombus and no evidence of structural heart disease. An adrenaline infusion was commenced with some initial improvement in blood pressure and heart rate. Cranial ultrasound showed bilateral intraventricular thrombus. III:3 developed intractable supraventricular tachycardia and the electroencephalogram became isoelectric. Intensive care was discontinued and III:3 died at 17 h of age. Placental histopathology showed some evidence of maternal and fetal mal-perfusion and chorioamnionitis, but no growth was observed in cultures. A chromosome microarray was normal.

III:4: the pregnancy with III:4 was uneventful. A cardiac review by ultrasound at 15 weeks gestation and a fetal morphology ultrasound at 19 weeks gestation were both normal. Additional ultrasound scans at 22- and 26-weeks gestation showed normal fetal growth and wellbeing. At 27 + 6 weeks gestation the mother presented with ruptured membranes. Steroids, magnesium sulfate, and antibiotics were commenced. Cultures of high vaginal swab samples showed *E. Coli* growth and antibiotics were adjusted accordingly. The mother went into spontaneous labor and male proband III:4 was born by emergency Cesarean section at 28 + 3 weeks gestation for bulging membranes and footling breech presentation. The arterial cord pH was 7.28 and lactate 5.6 mmol/l. At birth, III:4 appeared in good condition with 1 and 5 min Apgar scores of 8 and 9, respectively. The birth weight was 1295 g (78th percentile) and the head circumference was 27.5 cm (90th percentile). He was admitted to the neonatal intensive care unit for prematurity and was commenced on antibiotics. Blood cultures were subsequently negative. III:4 was commenced on continuous positive airway pressure and over several hours had increased respiratory effort and oxygen requirement. At 6 h of age, he was intubated and given surfactant for Hyaline Membrane disease. At 7 h of age, he became pale, floppy and had episodic desaturation. Blood gases showed a rapidly progressive severe metabolic acidosis. A cardiac ultrasound showed an almost non-contractile heart with DCM, as well as low cardiac output associated with aortic valve incompetence and dilated inferior vena cava. There was no evidence of coarctation or structural cardiac anomalies. III:4 did not respond to maximal support with intubation, ventilation, inotropes, and bicarbonate infusion and he died at 9 h of age.

### Genetic testing

Trio exome studies were performed on III:4 and his parents (II:3 and II:4). A homozygous variant in *LMOD2*, (GRCh38)chr7:g.123656237G > A; NM_207163.2:c.273 + 1G > A (ClinVar accession number: SCV002038515), was identified, which was predicted to abolish the donor 5′ splice-site of intron-1 (see in silico analysis in Supplementary Fig. II). Sanger segregation of this variant in family members confirmed that c.273 + 1G > A segregated with disease: III:3 and III:4 were homozygous, both parents and two healthy siblings were heterozygous and one healthy sibling had wild-type alleles (Fig. 1A). Additional members of the extended family were not available for genetic testing.

### Pathology

A full post-mortem examination found III:4 to be a normally grown 28-week gestation baby with no dysmorphic features. The heart was slightly increased in weight (10.8 g, normal: 7.3 ± 1.9 g) with evidence of biventricular dilatation (left significantly more than right, and the right ventricle was slightly shorter than the left, Fig. 1B). All valve rings were also dilated. The aorta was normal with no evidence of coarctation or interruption. Apart from cardiac pathology, no other abnormalities were identified. Microscopy of the heart muscle showed normal arrangement and architecture of myofibres (haematotoxylin and eosin staining, not shown). Thin filament structure and length in cardiac tissue of III:4 was examined using phalloidin (actin filament), tropomodulin-1 (thin filament pointed end) and α-actinin (Z-disc) staining (Fig. 1C). Despite normal spacing of α-actinin (~1.8–2.2 µm, see scale bar), the actin filaments labeled by phalloidin were drastically shortened/malformed in III:4 (0.220 µm ± 0.0403) compared to a 14-month-old control (0.995 µm ± 0.0579) and mice at day 12.5 of embryonic development reported in the literature (~0.77 µm) [8].

### The *LMOD2* c.273 + 1G > A variant disrupts splicing and *LMOD2* expression

**Fig. 2:**
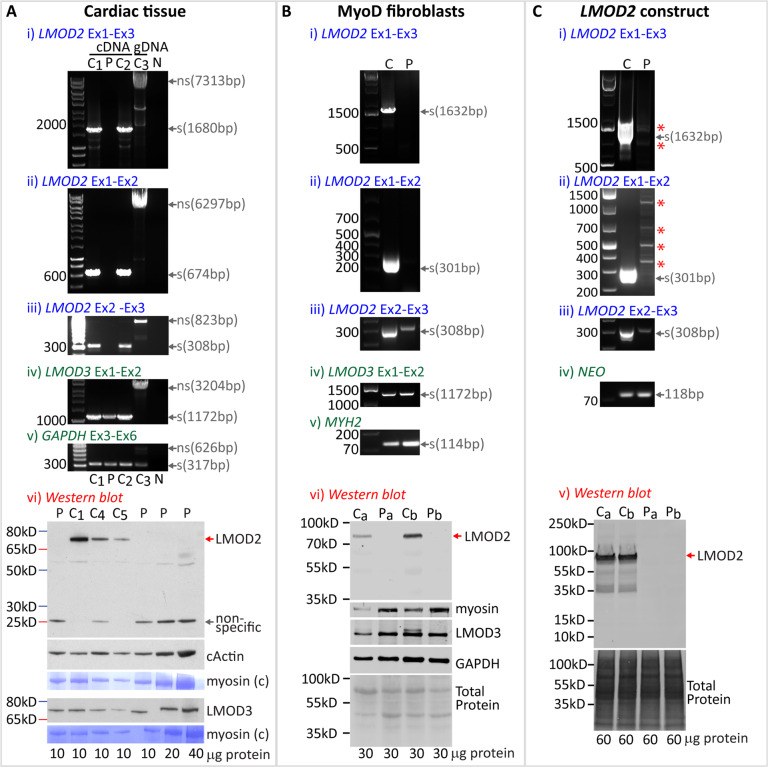
*LMOD2* c.273 + 1G > A causes a lack of canonically spliced exon 1-2-3 transcripts and full-length LMOD2 protein. **A**, **B**i–v, **C**i–iv RT-PCR to assess *LMOD2* pre-mRNA splicing in **A** cardiac tissue; **B** MyoD-fibroblasts; and **C** HEK293 cells transfected with an *LMOD2* gene construct (III:4 proband sample or c.273 + 1G > A gene construct [P] and control samples or wild-type gene construct [C]). Primer sets targeting the area of (i) exon 1-2, (ii) exon 1-3 and (iii) exon 2-3 were used for amplification. Bands, not corresponding to the predicted size of a canonically spliced product, were detected in HEK293 cells transfected with the *LMOD2* c.273 + 1G > A variant construct (**C**i and **C**ii, red asterisks; shown in Supplementary Fig. IV to correspond to cryptic splice-site use). Successful amplification of *LMOD3* exon 1-2 (**A**, **B**iv), *GAPDH* exon 3-6 (**A**v), *MYH2* (**B**v) and Neomycin (**C**iv, *NEO*, part of the *LMOD2* gene construct) served as positive controls for the PCR. LMOD2 Western blots in (**A**vi) cardiac tissue, (**B**vi) MyoD-fibroblasts and (**C**v) HEK293 cells transfected with an *LMOD2* gene construct (wild-type [C_a_, C_b_] or c.273 + 1-G > A [P_a_, P_b_]). (C_a_, C_b_, P_a_, P_b_) denotes samples from two independent experimental repeats. The position of full-length LMOD2 protein is indicated by a red arrow [predicted molecular weight: 61 kD], migrates at ~70 kD due to highly charged N-terminal region). A ~28 kD band detected in cardiac tissue of III:4 and C_4_ (gray arrow) is also present in LMOD2 KO mouse heart (Supplementary Fig. VI) indicating it is a non-specific band. Western blot of LMOD3, cardiac actin and Coomassie-stained myosin served as loading controls in cardiac tissue (**A**vi). In cell culture models, equal loading is shown with total protein stain (Ponceau S staining; **B**vi and **C**v) and successful myogenic conversion with myosin and LMOD3. The amount of protein lysate (in µg) is indicated below each lane. Controls: C_1_ (female, 10 years), C_2_ (female, 8 months), C_3_ (blood gDNA, female, 33 years), C_4_ (male, 2 years), C_5_ (unknown gender and age). C_4_ and C_5_ are human ventricular myocardium obtained as part of corrective surgery for children with structural and congenital heart disease. Note: neonatal control samples were not available for analysis. However, LMOD2 protein is detectable in perinatal/neonatal mouse left ventricular lysates [8] and MyoD-fibroblasts (a model for immature muscle); and RNAseq data reveals prenatal *LMOD2* mRNA expression in humans [22]. exon (Ex) intron (In).

RT-PCR was performed on cDNA derived from a III:4 cardiac autopsy tissue sample and cardiac control tissue to determine the effect of the *LMOD2* c.273 + 1G > A variant on *LMOD2* transcription and pre-mRNA splicing (Fig. 2Ai–v). RT-PCR of three control genes (two muscle specific [*PYGM* and *LMOD3*] and one ubiquitously expressed gene [*GAPDH*]) resulted in less amplicon in the patient than in two control samples (C_1_ and C_2_; see Supplementary Fig. IIIA). We confirmed that this was due to reduced quality of III:4 cardiac tissue RNA (Supplementary Fig. IIIB, RINe 5.8). Therefore, in an attempt to normalize for this, we used five times the amount of cDNA from III:4 compared to controls (25 ng versus 5 ng) and the PCR cycles were increased from 30 to 35 cycles. These parameters enabled robust amplification of three control genes (*PYGM, LMOD3, GAPDH*) in III:4, but failed to amplify amplicons corresponding to *LMOD2* exons 1-2-3 (Fig. 2Ai), *LMOD2* exons 1-2 (Fig. 2Aii) or *LMOD2* exons 2-3 (Fig. 2Aiii). Both controls showed amplicons of the predicted sizes. Of note, amplification of *LMOD3* exons 1-2 (another leiomodin expressed in striated muscles, Fig. 2Aiv) and *GAPDH* exons 3-5 (Fig. 2Av) was similar in III:4 and control samples, consistent with specific loss of canonically spliced *LMOD2* transcripts.

To overcome interpretative caveats related to RNA decay observed in the cardiac autopsy sample from III:4, we conducted studies of *LMOD2* pre-mRNA splicing in two cell culture models: (1) skin fibroblasts from III:4 and a control, transduced with Adv-MyoD to force myogenesis (MyoD-fibroblasts, see Supplementary Fig. I) [15]; and (2) HEK293 cells transfected with a wild-type (c.273 + 1G) or variant (c.273 + 1G > A) *LMOD2* gene construct.

In control MyoD-fibroblasts, PCR amplicons corresponding to canonical splicing of *LMOD2* exons 1-2-3 (Fig. 2Bi) and *LMOD2* exons 1-2 (Fig. 2Bii) were robustly amplified, while they were absent in III:4 MyoD-fibroblasts. Importantly, PCR amplicons for muscle-specific proteins *LMOD3* (Fig. 2Biv) and *MYH2* (Fig. 2Bv) confirmed induced myogenesis for both specimens. Similarly, transfection of HEK293 cells with the wild-type *LMOD2* gene construct (c.273 + 1G) produced abundant levels of an amplicon corresponding to canonical splicing of *LMOD2* exons 1-2-3 (Fig. 2Ci). In contrast, we found no evidence for *LMOD2* transcripts with canonical splicing of exons 1-2-3 arising from the variant construct (c.273 + 1G > A), and instead amplified multiple, faint bands (c.273 + 1G > A; Fig. 2Cii, asterisks). Cloning and Sanger sequencing identified these amplicons as *LMOD2* gene products spliced at alternative (cryptic) splice-sites (schematic representation in Supplementary Fig. IV).

**Fig. 3:**
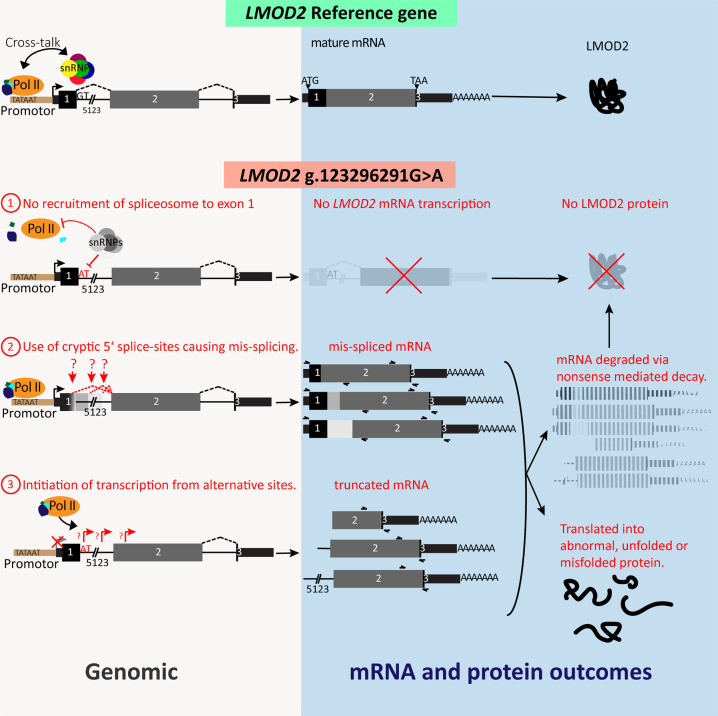
Proposed pathogenic mechanism resulting in loss of transcripts and protein from *LMOD2* GRCh38)chr7:g.123656237G > A; NM_207163.2:c.273 + 1G > A. Potential mechanistic causes resulting in loss of *LMOD2* transcripts with canonical splicing of exons 1-2-3. (1) Lack of spliceosomal recognition and assembly at the c.273 + 1G > A variant donor results in loss of important crosstalk between the RNA polymerase (PolII) and early spliceosome that can result in stalled or terminated transcription. (2) Transcript correctly initiating at exon-1 are spliced using weak exonic and intronic cryptic donor splice-sites (indicated by “?”). Cryptic donor(s) detected result in an encoded premature termination codon potentially eliciting nonsense-mediated decay. (3) Aberrant initiation or elongation of transcription may activate use of an alternative transcription start site. This results in start-loss transcripts either targeted by nonsense-mediated decay or encode a truncated LMOD2 protein.

Interestingly, a PCR amplicon confirmed by Sanger sequencing to correspond to *LMOD2* transcripts spliced from exon 2-3 was detectable in both cell models (Fig. 2Biii, Ciii). The amplicon band was weaker in proband MyoD-fibroblasts, and in HEK293 cells transfected with the *LMOD2* c.273 + 1G > A gene construct, compared to their respective controls (Fig. 2Biii, Ciii and Supplementary Fig. V). The detection of low abundance, alternatively spliced Exon 1-2-3 amplicons, and yet higher abundance, normally spliced Exon-2-3 amplicons, could indicate variant-induced activation of an alternative transcription start-site downstream of exon-1 and upstream of the primer in exon-2: a known consequence of variants ablating the intron-1 donor [16, 17] (see Fig. 3 and “Discussion”).

### The *LMOD2* c.273 + 1G > A variant results in absence of full-length *LMOD2*

Western blot analysis for LMOD2 was performed on III:4 cardiac tissue lysate, III:4 MyoD-fibroblasts and HEK293 cells transfected with *LMOD2* gene constructs (Fig. 2Avi, Bvi, Cv). Full-length LMOD2 migrating at ~70 kDa was detected in all respective controls but was absent in III:4 cardiac tissue lysate, III:4 MyoD-fibroblasts and *LMOD2* c.273 + 1G > A transfected HEK293 cells. A ~28 kDa protein product was detected in III:4 cardiac tissue which was also present in cardiac control C_4_. This band likely represents a non-specific protein recognized by the antibody since a protein of similar size is detected in cardiac tissue from the *Lmod2* KO mouse (Supplementary Fig. VI). In cardiac tissue, levels of LMOD3 and cardiac actin in the proband sample were similar to controls, with no evidence of proteolytic degradation, providing supporting evidence that LMOD2 deficiency in III:4 is not caused by tissue decay. In MyoD-fibroblasts, robust expression of LMOD3 and myosin heavy chain served as myogenic differentiation controls (Fig. 2Bvi). GAPDH (Fig. 2Bvi) and total protein stain confirmed equal protein loading in MyoD-fibroblasts and transfected HEK293 cells.

## Discussion

This study describes two siblings who are homozygous for a novel *LMOD2* variant ablating the donor splice-site of intron-1 (NM_207163.2:c.273 + 1G > A). Evidence from *LMOD2* pre-mRNA splicing studies showed that the c.273 + 1G > A variant results in undetectable levels of *LMOD2* transcripts with canonical exon 1-2-3 splicing. The likely mechanisms leading to a lack of *LMOD2* transcripts are depicted in Fig. 3. One explanation relates to variant-associated defects in transcription initiation or elongation due to loss of the intron-1 donor (mechanism 1 in Fig. 3). As the RNA polymerase passes over the intron-1 donor, there is a transcription checkpoint regulated by crosstalk between the RNA polymerase and early spliceosome assembling at the intron-1 donor [16, 17]. When this does not occur, the RNA polymerase can stall and/or transcription can terminate.

Another possible mechanism relates to the use of cryptic splice-sites instead of the mutated canonical splice-site (mechanism 2 in Fig. 3). We detected low abundance splicing events using cryptic donors in exon-1 and intron-1 in the HEK cell culture systems which were not detectable in cardiac tissue and MyoD-fibroblasts from the proband. This different RT-PCR outcome in HEK293 cells is most likely caused by the expression of *LMOD2* above physiological expression levels in this model system (driven by a cytomegalovirus promotor), resulting in easier detection of low abundance products and/or less efficient removal of aberrant transcripts via nonsense-mediated decay.

Despite not amplifying any product from III:4 MyoD-fibroblast RNA with numerous exon-1 primers, we amplified a band corresponding to splicing of *LMOD2* exons 2-3 in both cell culture models. In HEK293 cells it is possible that these amplicons originate from exon-1 (miss-spliced exon 1-2-3 amplicons were also detected). In MyoD-transduced fibroblasts there was no evidence that these transcripts initiated at exon-1, consistent with potential variant-induced activation of an alternative transcription start site downstream of exon-1 (mechanism 3 in Fig. 3). *LMOD2* transcripts that do not initiate at exon-1 lack the encoded start methionine and are akin to a start-loss variant.

In summary, there appear to be multiple mechanisms potentially at play resulting in absence of canonically-spliced (exons 1-2-3) *LMOD2*: (1) impaired initiation or elongation of transcription; (2) spliceosomal use of weak cryptic exon-1/intron-1 donor splice-sites or (3) activation of alternative transcription start-site (mechanism (2) and (3) likely result in nonsense-mediated decay targeted aberrant *LMOD2* transcripts encoding a premature termination codon).

Consistent with the RT-PCR results, *LMOD2* c.273 + 1G > A was associated with loss of full-length LMOD2 protein in cardiac tissue and MyoD-fibroblasts from the affected proband, with gene construct transfection assays proving evidence that loss of expressed LMOD2 protein results specifically from the *LMOD2* c.273 + 1G > A substitution.

It is important to note that we used available antibodies which all target the N-terminus of LMOD2 and thus cannot exclude the presence of truncated LMOD2 proteins originating from the exon 2-3 transcript (detected in both cell models but not in tissue) or miss-spliced exon 1-2-3 transcripts (detected only in HEK293 cells) and translated using alternative start codons. If N-terminally truncated LMOD2 is indeed made, it is likely functionally impaired as it lacks critical N-terminal actin and tropomyosin binding domains [18, 19]. The presented data are consistent with loss of full-length LMOD2 as the primary basis for the DCM in two infants bearing the *LMOD2* c.273 + 1G > A variant, with any truncated LMOD2 proteins (if present) unable to function normally.

This study confirms the role of *LMOD2* loss-of-function variants as a cause for an autosomal recessive, neonatal-onset, lethal DCM which segregates as an autosomal recessive disorder. A previous report describes a female neonate with DCM associated with a homozygous nonsense variant in *LMOD2* exon-2 (c.1193G > A;p.Trp398*) [6]. Similar to the splice-site variant described here, c.1193G > A; p.Trp398* resulted in absence of LMOD2 protein by western blot. The clinical and histological phenotype reported in Ahrens-Nicklas et al. [6], is highly concordant with the two infant siblings presented herein and is mirrored by two *Lmod2*-deficient mouse models; onset at birth leading to cardiac ventricular dilatation and a non-contractile heart leading to heart failure, with associated shortening of cardiac thin filaments [6–8]. While both siblings presented herein died within hours of birth, maximal support via extracorporeal membrane oxygenation and a Berlin Heart left ventricular assist device sustained the female infant reported in [6] until a heart transplant became available at 10 months of age.

Why cardiac abnormalities associated with *LMOD2* loss-of-function variants are not detected prior to birth and cardiac function rapidly declines following birth are currently incompletely understood and warrant further investigations. *Lmod2* KO mouse studies showed thin filament shortening prior to birth and prior to onset of cardiac symptoms [8]. After birth, the heart undergoes a dramatic change in hemodynamics, including an increase in systemic vascular resistance. We hypothesize, that the resulting increase in cardiac afterload/preload stretches the cardiac ventricles, leading to longer end-diastolic sarcomere lengths. Since the thin filaments are shorter, the thin/thick filaments overlap is reduced or absent at long sarcomere lengths resulting in reduced contractile force and ejection fraction (also discussed in [8]). An alternative hypothesis is that another protein (such as LMOD3) compensates for the loss of LMOD2 prenatally. Further studies are required to investigate this possibility; however, we currently have no evidence that LMOD2 and LMOD3 have overlapping functions. *Lmod3* KO mice and patients with loss-of-function variants in LMOD3 have no cardiac phenotype suggesting a critical role of LMOD3 in skeletal muscle only [9, 20].

In conclusion, we identified the first pathogenic splice-site variant in *LMOD2* confirming it as an etiology for autosomal recessive DCM and a critical protein in the dynamic regulation of thin filament length in the heart. This study highlights the importance of considering splice-altering coding or non-coding variants in *LMOD2* for undiagnosed cases of severe, neonatal DCM of unknown etiology.

## Supplementary information

Supplemental Material

## Data Availability

Additional data are available from the corresponding author on reasonable request.
