# Supplementary material for: Neonatal-lethal dilated cardiomyopathy due to a homozygous LMOD2 donor splice-site variant
Source: Eur J Hum Genet. 2022 Jan 26;30(4):450–7. doi: 10.1038/s41431-022-01043-8 (PMC8989920; doi:10.1038/s41431-022-01043-8)
Supplement: Supplementary file 1 — Supplemental Material [file 41431_2022_1043_MOESM1_ESM.docx]

# SUPPLEMENTAL MATERIAL

**Neonatal-lethal dilated cardiomyopathy due to a homozygous *LMOD2* donor splice-site variant.**

Michaela Yuen PhD^1,2*^, Lisa Worgan MBBS^3*^, Jessika Iwanski MS^4^, Christopher T. Pappas PhD^4^, Himanshu Joshi^1^, Jared M. Churko PhD^4^, Susan Arbuckle MD^5^, Edwin P. Kirk MBBS PhD^6,7,8^, Ying Zhu^6^, Tony Roscioli FRACP, PhD^6,8,9^, Carol C. Gregorio PhD^4Ɨ^, and Sandra T. Cooper PhD^1,2,10 Ɨ^

^1^Kids Neuroscience Centre, The Children's Hospital at Westmead, Westmead, NSW, Australia;

^2^Discipline of Child and Adolescent Health, Sydney Medical School, University of Sydney, Camperdown, NSW, Australia;

^3^Department of Medical Genomics, Royal Prince Alfred Hospital, Camperdown, NSW, Australia;

^4^Department of Cellular and Molecular Medicine, and Sarver Molecular Cardiovascular Research Program, The University of Arizona, Tucson, AZ, United States;

^5^Department of Histopathology, The Children's Hospital at Westmead, Westmead, NSW, Australia;

^6^New South Wales Health Pathology Randwick Genomics Laboratory, Randwick, NSW, Australia;

^7^School of Women’s and Children’s Health, University of New South Wales, Randwick, NSW, Australia;

^8^Centre for Clinical Genetics, Sydney Children’s Hospital, Randwick, NSW, Australia

^9^Neuroscience Research Australia (NeuRA), University of New South Wales, Sydney, NSW, Australia;

^10^The Children’s Medical Research Institute, Westmead, NSW, Australia

**MY and LW have contributed equally to this work*

*^Ɨ^CCG and STC have contributed equally to this work*

**Correspondence:**

Michaela Yuen, PhD; Kids Neuroscience Centre, The Children's Hospital at Westmead; michaela.yuen@sydney.edu.au

## Supplemental methods

### PCR cycling conditions

***Cardiac tissue.*** Cycling conditions for Taq DNA Polymerase were 95 °C for 2 min followed by 25 or 35 cycles of 95°C for 10 s, 60°C or 64°C (see **Supplemental Table I**) for 30 s, 72 °C for 90 s. Final extension was done for 8 min at 72 °C. Cycling conditions for LongAmp® Taq DNA Polymerase were 94 °C for 2 min followed by 25 or 35 cycles of 94 °C for 15 s, 60 °C or 64 °C (see **Supplemental Table I**) for 30 s, 65 °C for 3-6min (depending on amplicon length, ~50 s/kb). Final extension was performed for 8 min at 65 °C.

***MyoD-fibroblasts.*** Cycling conditions for GoTaq® DNA Polymerase were 95 °C for 2 min followed by 29 (Lmod3), 33 (Myh2), 35 (Lmod2 Ex2-3), 40 (Lmod2 Ex1-3) and 44 (Lmod2 Ex1-2) cycles of 95 °C for 30 s, 60 °C or 64 °C (see **Supplemental Table I**) for 30 s, 72 °C for 30s, 1min, 2.5 min or 6 min (depending on amplicon length). Final extension was performed at 72°C for 5 min. Cycling conditions for LongAmp® Taq DNA Polymerase were 94 °C for 2 min followed by 40 cycles of 94 °C for 15s, 64 °C for 30s, and 65 °C for 6min (see **Supplemental Table I**). Final extension was performed at 65 °C for 10 mins.

***HEK293 cells.*** Cycling conditions for GoTaq® DNA Polymerase were 95 °C for 2 min followed by 26 (Lmod2 Ex2-3), 30 (Neo), or 38 (Lmod2 Ex1-2-A,B) cycles of 95 °C for 30 s, 60 °C (see **Supplemental Table I**) for 30 s, 72 °C for 50s or 6 min (depending on amplicon length). Final extension was performed at 72°C for 5 or 10 min.

## Supplemental figures and tables

**Supplemental table I: Primers and PCR conditions**

| **Primer name** | **Sequence 5'-3'** | **Product ns/s (bp)** | **Annealing temperature (°C)** | **Extension time** | **Description** |
| --- | --- | --- | --- | --- | --- |
| *LMOD2*_E1_FW *LMOD2*_E3_RW | AGCTCCTTCTGGGTCTGACA TGTTTTTATCGCAGGGCTTC | 7313/1680 | 64 | 6min | Figure 2Ai; Ex1 to Ex3 |
| LMOD2_E1_2_FW LMOD2_E3_2_RW | TGGCTACCGAAGAGGACTCA TATCGCAGGGCTTCTGGAAC | 7770/1632 | 64 | 6min | Figure 2Bi and 2Ci; Ex1 to Ex3 |
| LMOD2_E1_FW LMOD2_E2_RW | AGCTCCTTCTGGGTCTGACA CTGTGGTGTCAGGGTCATTG | 6297/674 | 64 | 6min | Figure 2 Aii; Ex1 to Ex2 |
| LMOD2_E1_2A_FW LMOD2_E2_2A_RW | TGGCTACCGAAGAGGACTCA GATAAGCTCTTCTTCACTTTCCTC | 5924/301 | 60 | 6min | Figure 2Bii and 2Cii; Ex1 to Ex2 |
| LMOD2_E1_2B_FW LMOD2_E2_2B_RW | GGACATTCAGCAGAGAGGCACT  GATAAGCTCTTCTTCACTTTCCTC | 5751/128 | 60 | 6min | Supplemental Figure IV; Ex1 to Ex2 |
| LMOD2_E2_FW LMOD2_E3_RW | CCTCCTCCACTCCCAGAGAA TATCGCAGGGCTTCTGGAAC | 823/308 | 67 | 60s | Figure 2A-Ciii and Supplemental Figure V; Ex2 to Ex3 |
| *LMOD3_*E1_FW *LMOD3_*E2_RW | CATGCTGGAAGAGGAACGAG GCGATGGCTTTTTCATCATT | 3204/1172 | 60 | 2.5min | Figure 2A-Biv; Ex1 to Ex2 |
| *GAPDH*_Ex3_FW *GAPDH*_Ex6_RW | TCACCAGGGCTGCTTTTAAC GGCAGAGATGATGACCCTTT | 626/317 | 64 | 90s | Figure 2Av; Ex3 to Ex6 |
| *PYGM* Ex1 FW *PYGM* Ex8 RW | CTCGTAAAGGACCGCAATGT GACTTGAAGCGACGGATGAT | 5031/824 | 64 | 6min | Supplemental Figure III; Ex1 to Ex8 |
| *MYH2*_FW *MYH2*_RW | GGAGGACAAAGTCAACACCCTG GCCCTTTCTAGGTCCATGCGAA | 196/114 | 64 | 30s | Figure 2Bv |
| *NEO*_FW *NEO*_RW | GATGGATTGCACGCAGGTTC  TCAGAGCAGCCGATTGTCTG | 86 | 60 | 50s | Figure 2Civ |
| Abbreviations: ns = not spliced, s = spliced, Ex = exon, bp = base pairs | | | | | |


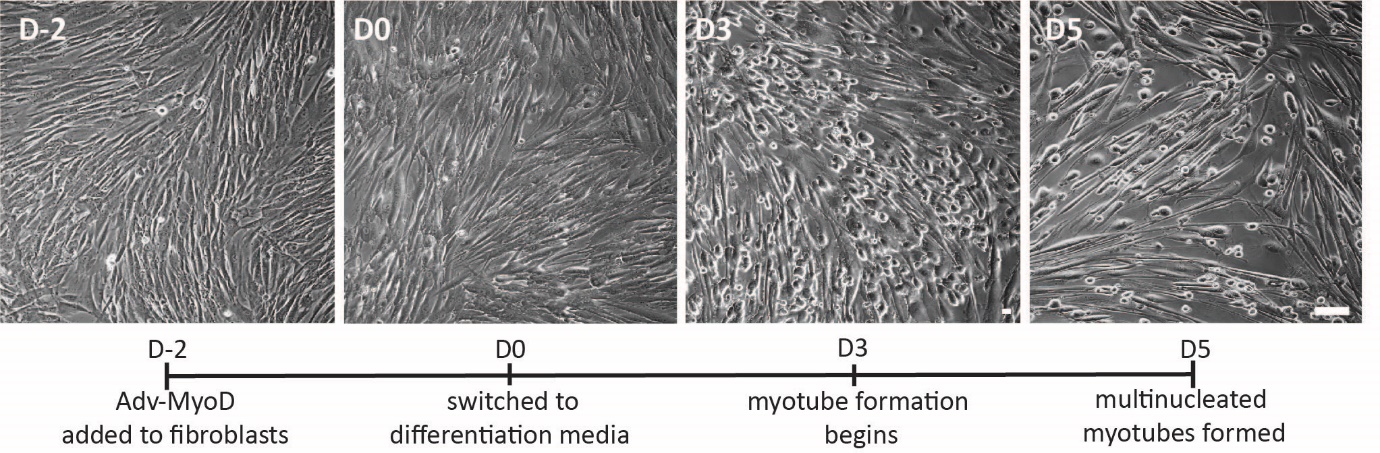


**Supplemental figure I. Direct reprogramming of human primary skin fibroblasts into myotubes.**

Fibroblasts were seeded onto a Matrigel-coated (1:100) (Fisher) 6-well plate at 80-90% confluency and transduced with human adenovirus type 5(dE1/E3) expressing MyoD (Ad-MyoD, Vector Biolabs) at a multiplicity of infection (MOI) of 200 (**D-2**). Differentiation of fibroblasts into myotubes was induced 2 days after adenoviral transduction by replacing culture media with differentiation media (**D0**) containing 3% horse serum (Gibco), 1% Insulin Transferrin-Selenium-A (Gibco), and gentamicin solution (Sigma) in DMEM/F12. Media was replaced daily. MyoD-transduced skin fibroblasts underwent myogenesis and were observed to first form myotubes after 3 days in differentiation media (**D3**), with multinucleated myotubes forming on differentiation day 5 (**D5,** myotubes were collected for RNA and protein studies). Scale bar = 10 μm.


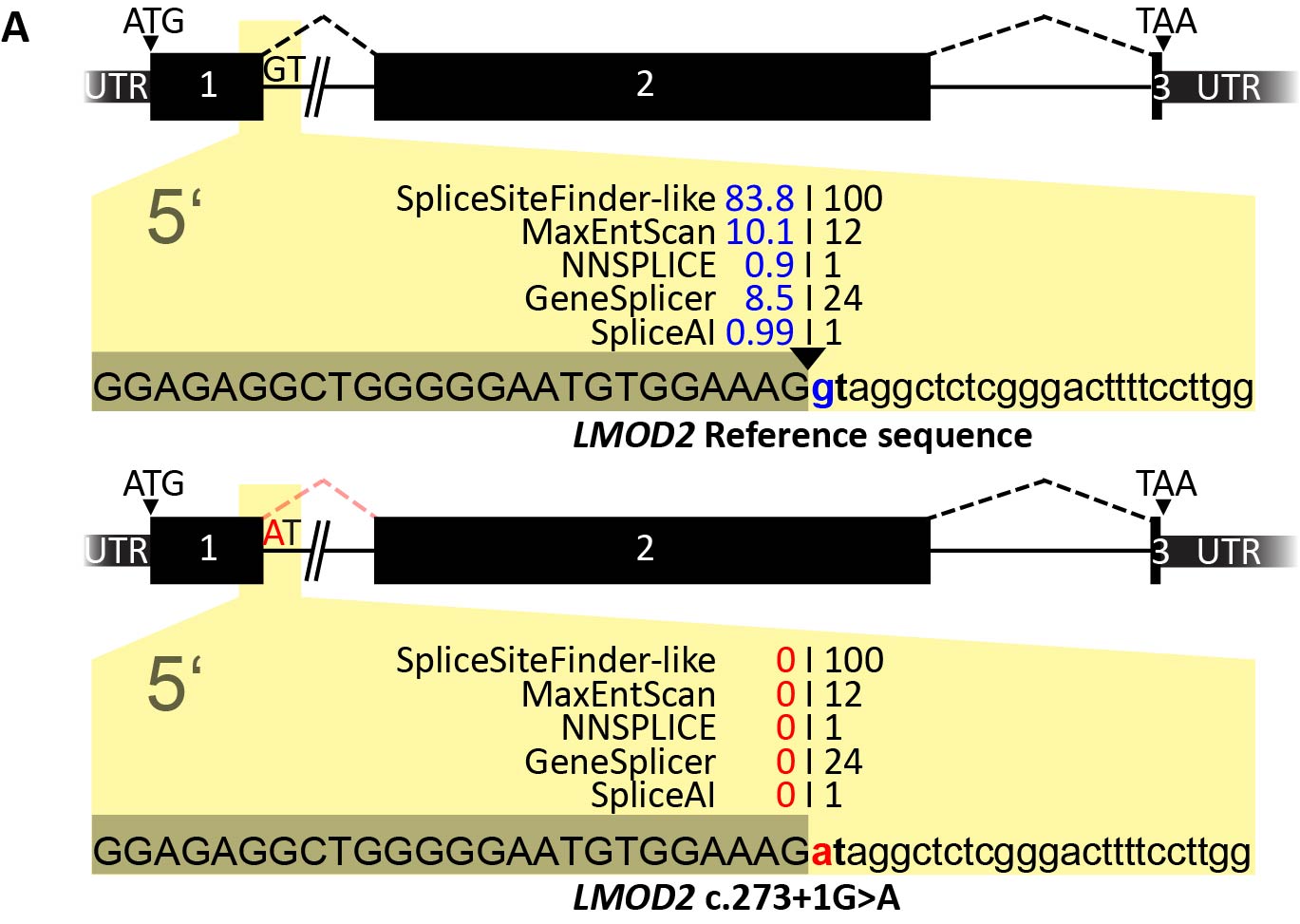


**Supplemental figure II. *In silico* splicing prediction algorithms suggesting the LMOD2 c.273+1G>A variant ablates the 5’ splice-site of intron-1.**

*In silico* splicing analysis of the index variant was performed using Alamut Visual® v.2.12 rev.0 (Interactive Biosoftware, Rouen, France). *LMOD2* gene structure based on NM_207163 with the coding sequence (start ATG and stop codon [TAA] annotated) shown as black squares and introns as lines (note: intron-1 was shortened, indicated by parallel lines). *LMOD2* consists of three exons and 2 introns flanked by 5’ and 3’ untranslated regions (UTR). The 5’ untranslated region (UTR) and start methionine is encoded by exon-1, the vast majority of the open reading frame is encoded by exon-2, the termination codon and long 3’UTR is encoded by exon-3. The reference sequence with the affected nucleotide highlighted in blue is shown on top. Splice-site prediction tools Alamut Visual® (integrated algorithms: SpliceSiteFinder-like, MaxEntScan, NNSPLICE and GeneSplicer) and SpliceAI both suggest the presence of a splice-site before the affected nucleotide (scores in blue; eg. SpliceSiteFinder-like predicts splicing with a score of 83.8 of 100). The *LMOD2* c.273+1G>A variant ablates this donor splice-site (variant shown in red at bottom, along with prediction scores of 0 in red).

**
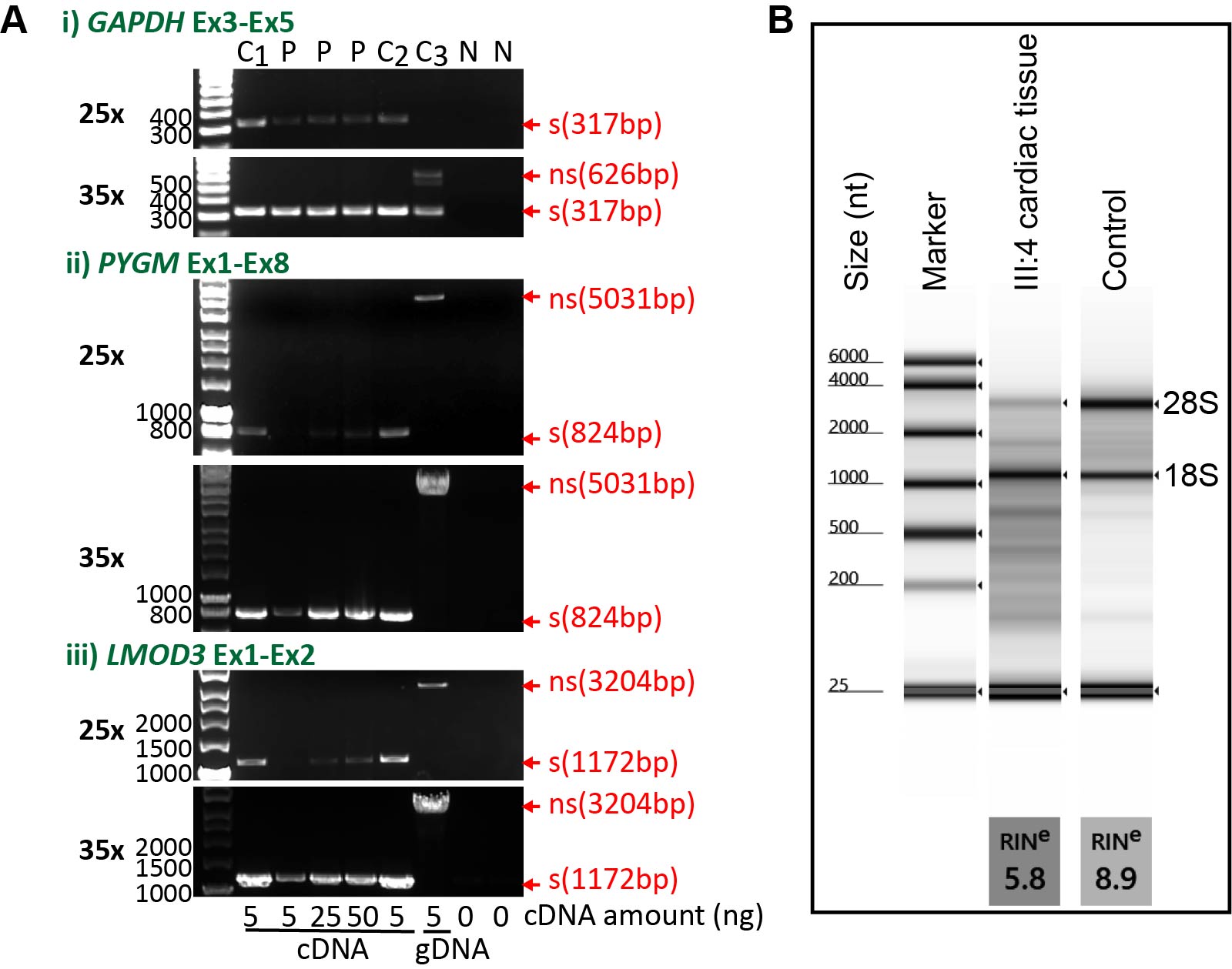
**

**Supplemental figure III. Amplification of control genes suggest poor quality of template RNA extracted from III:4 cardiac tissue.**

(**A**) Amplification of (**i**) *GAPDH* (ubiquitously expressed), (**ii**) *PYGM* (muscle specific) and (**iii**) *LMOD3* (another muscle specific member of the leiomodin family) resulted in a weaker amplicon band in the patient suggesting lower quality/quantity of RNA extracted from proband III:4 cardiac tissue than RNA extracted from control cardiac tissue (C_1_ and C_2_; when comparing reactions containing 5 ng of template cDNA). This is particularly apparent after 25 PCR cycles. 25 ng of patient cDNA and 5 ng of control cDNA resulted in a similar amount of amplification in patient and control samples after 35 PCR cycles. These conditions were used for *LMOD2* PCRs shown in **Figure 2B**. (**B**) Agilent TapeStation RNA analysis of the 18s and 28s ribosomal RNAs confirmed reduced RNA quality of proband III:4 (RNA integrity number equivalent [RINe] of 5.8). For comparison, an RNA sample isolated from a better-preserved muscle specimen is shown in the last lane (control, RINe of 8.9).


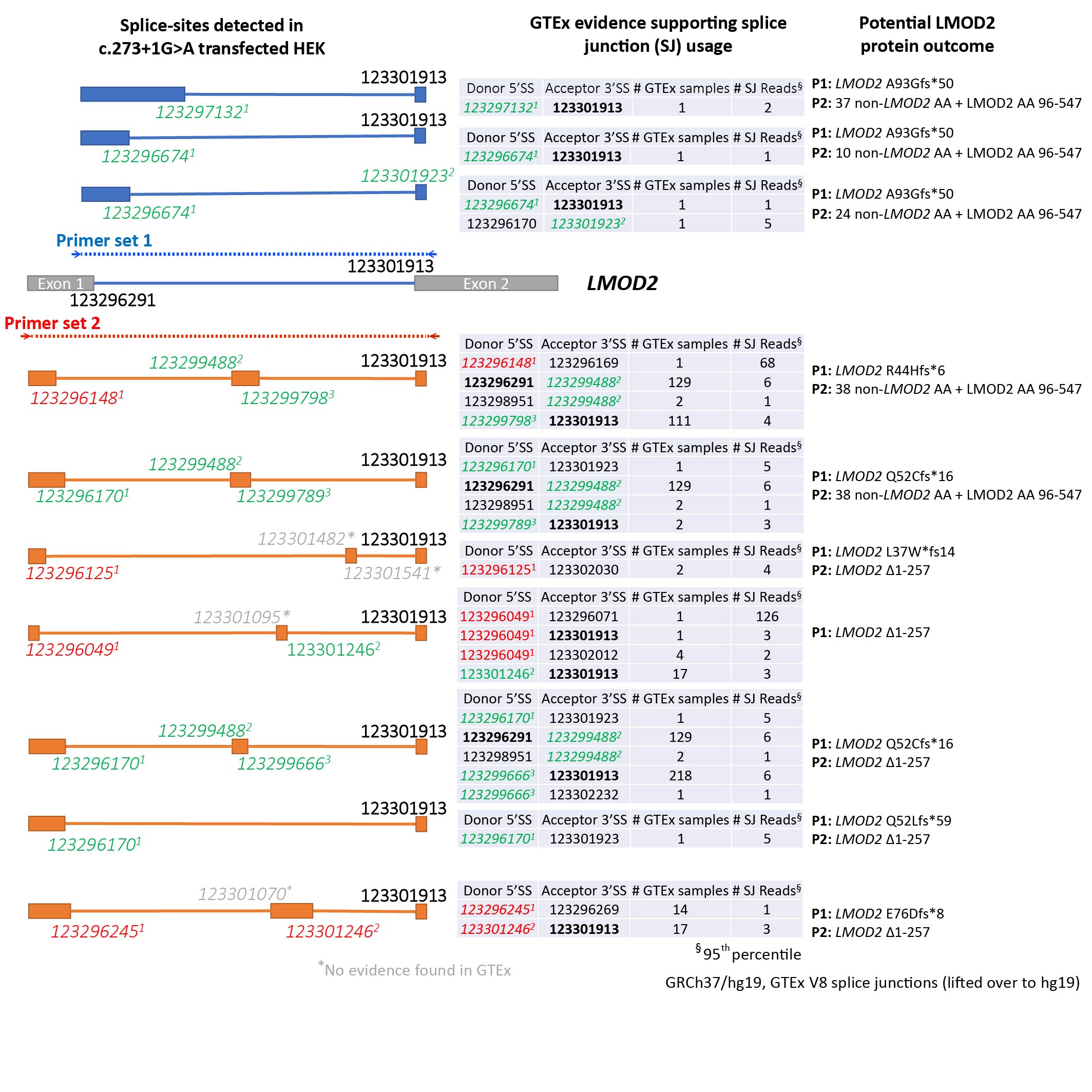


**Supplemental figure IV. Splice junctions identified in *LMOD2* c.273+1G>A transfected HEK293 cells**

cDNA obtained from *LMOD2* c.273+1G>A transfected HEK293 cells was amplified with two primer sets (blue – primer set 1; orange – primer set 2). Amplicons were cloned, sequenced and sequences were aligned to Chromosome 7 (NC_000007.13) using NCBI Genome Workbench^1^; BLASTn: Word size: 7, e-value 20, Low complexity regions filtered). Candidate cryptic donor and acceptor sites shown in the graphs on the left were identified from aligned sequences using IGV.^2^ The usage and coordinates of splice junctions shown in tables on the right were elucidated using summarised GTEx splice junction read counts.^3^ Per sample, splice junctions were obtained from GTEx (phs000424.v8.p2). Splice junction read counts were summarised across all samples for each unique splice junction and translated from GRCh38 to GRCh37 using liftOver. Splice junction coordinates in bold correspond to the canonical splice junctions, while those shown in green are non-canonical splice junctions which were found to be supported by GTEx data. Coordinates printed in red are inexplicit (GTEx splice junction width not compatible with space constraints^4^. Splice junction is close to but not directly overlapping the fragment) and grey coordinates refer to positions which were not detected in the GTEx data set. Note: only the plus strand was considered for the analysis. Open reading frame (ORF) analysis was performed to determine potential proteins resulting from mis-spliced transcripts using ORFfinder (<https://www.ncbi.nlm.nih.gov/orffinder/>). Translation starting at the wild-type *LMOD2* start methionine is expected to result in short ORFs encoding the start of LMOD2 followed by a frame-shift due to abnormal splice-junction. A second, longer ORF was detected to be generated by all abnormally spliced HEK293 transcripts. This ORF encodes the C-terminus of LMOD2 starting from an alternative start codon and sometimes including non-*LMOD2* amino acids. This figure is based on data obtained from dbGaP accession number phs000424.v8.p2.


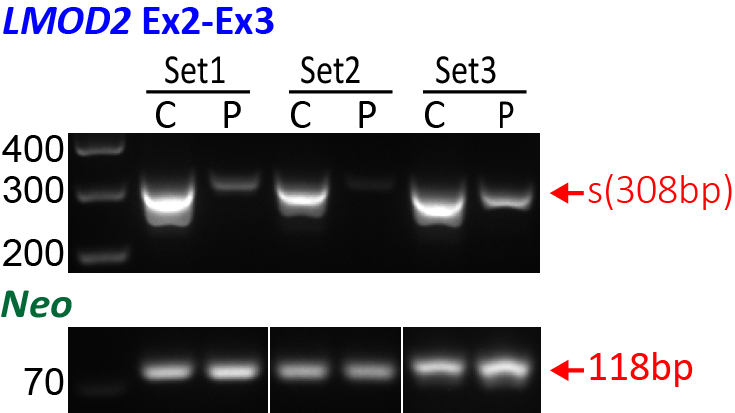


**Supplemental figure V. An amplicon spanning Exon-2 to 3 (Ex2-Ex3) is detected at reduced levels in HEK293 transfected with *LMOD2* c.273+1G>A construct relative to the reference c.273+1G control construct.**

Ex2-Ex3 PCR of three independent transfections of HEK293 cells with the *LMOD2* c.273+1G control construct (C) or the *LMOD2* c.273+1G>A construct (P) consistently show reduced amplicon levels in the variant construct (P). Neomycin (Neo) PCR shows equal amplification band intensity.


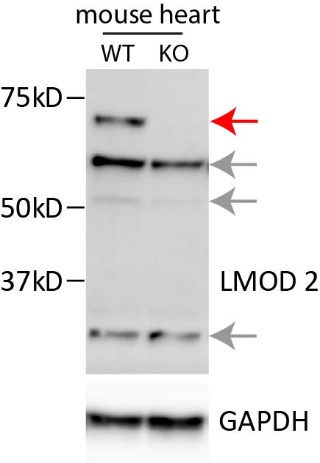


**Supplemental figure** **VI. Lmod2 detection by α-LMOD2 antibody (Santa Cruz, sc-135493, S-12) in wild-type (WT) and *Lmod2* knock-out (KO) mice**

10 µg of WT and KO mouse heart tissue lysates were separated on a 10 % Bis-Tris precast gel, transferred onto a PVDF membrane and probed with the S-12 α-LMOD2 antibody. A specific band is detected at about 70 kD (red arrow) in WT heart that is absent in KO heart. Several non-specific bands are also detected (grey arrows).

**References:**

1. Kuznetsov A, Bollin CJ. Ncbi genome workbench: Desktop software for comparative genomics, visualization, and genbank data submission. *Methods Mol. Biol.* 2021;2231:261-295

2. Robinson JT, Thorvaldsdottir H, Winckler W, Guttman M, Lander ES, Getz G, Mesirov JP. Integrative genomics viewer. *Nat. Biotechnol.* 2011;29:24-26

3. Consortium GT. The genotype-tissue expression (gtex) project. *Nat. Genet.* 2013;45:580-585

4. Bryen SJ, Joshi H, Evesson FJ, Girard C, Ghaoui R, Waddell LB, Testa AC, Cummings B, Arbuckle S, Graf N, Webster R, MacArthur DG, Laing NG, Davis MR, Luhrmann R, Cooper ST. Pathogenic abnormal splicing due to intronic deletions that induce biophysical space constraint for spliceosome assembly. *Am J Hum Genet*. 2019;105:573-587
